# Horizon scanning for European wild pollinators identifies world-leading legislation as a key opportunity for pollinators

**DOI:** 10.1038/s41598-026-53025-1

**Published:** 2026-05-15

**Authors:** Morgan A. Morrison, Anne Alix, Ignasi Bartomeus, Imre Demeter, Ciara Dwyer, Úna Fitzpatrick, Elena Gazzea, Costanza Geppert, Marina Janković Milosavljević, Reet Karise, David Kleijn, Zdravko Kolev, Simon G. Potts, Deepa Senapathi, Oliver Schweiger, Adam J. Vanbergen, Mark J. F. Brown

**Affiliations:** 1 Centre for Ecology and Conservation, University of Exeter, Cornwall, UK; 2 Department of Biological Sciences, Royal Holloway University of London, Egham, TW20 0EX UK; 3 European Policy and Global Precision Application Leader, 101 Park Drive, Milton Park, OX14 4RY UK; 4 Estación Biológica de Doñana (EBD-CSIC), Sevilla, Spain; 5 Lendület Ecosystem Services Research Group, Institute of Ecology and Botany, HUN-REN Centre for Ecological Research, Vácrátót, Hungary; 6 Centre for Environmental and Climate Science, Lund University, Lund, Sweden; 7 National Biodiversity Data Centre, Waterford, Ireland; 8 Department of Agronomy, Food, Natural Resources, Animals and Environment (DAFNAE), University of Padova, Viale dell’Università, 16, Legnaro, 35020 Padua Italy; 9 Department of Biology and Ecology, Faculty of Sciences, University of Novi Sad, Trg Dositeja Obradovića 2, Novi Sad, Serbia; 10 Chair of Plant Health, Institute of Agricultural and Environmental Sciences, Estonian University of Life Sciences, Tartu, Estonia; 11 Plant Ecology and Nature Conservation Group, Wageningen University & Research, Wageningen, The Netherlands; 12 National Museum of Natural History, 1 Tsar Osvoboditel Blvd, Sofia, 1000 Bulgaria; 13 Centre for Agri-Environmental Research, School of Agriculture, Policy and Development, Reading University, Reading, RG6 6AR UK; 14 Helmholtz Centre for Environmental Research-UFZ, 06120 Halle, Germany; 15 Université de Bourgogne Europe, Institut Agro, INRAE, Agroécologie, Dijon, 21000 France; 16 Department of Zoology, University of Cambridge, Cambridge, UK

**Keywords:** Ecology, Ecology, Environmental sciences

## Abstract

Wild insect pollinators contribute significantly to agricultural productivity, biodiversity, and ecosystem functioning. Wild pollinators are increasingly affected by multiple interacting stressors. Proactively identifying emerging risks and feasible mitigation strategies will be critical to ensuring the long-term stability of wild pollinators biodiversity and pollination services. We conducted the first continental scale horizon scan focused on wild pollinators in Europe. A structured Delphi-based approach was used to identify emerging issues that may have significant implications for wild pollinators over the coming decade. Ten priority issues were identified, including both potential risks and opportunities. For the first time in a pollinator-focused horizon scan, legislation was identified as a key opportunity, with the European Union Nature Restoration Regulation recognised for its potential to influence pollinator conservation through mandatory restoration and monitoring targets. In contrast, political developments such as the rise of populist parties and post-truth discourse may impede policy implementation. Several issues relating to pesticide use were also identified, including developments in RNA interference technologies and precision application methods, which may reduce non-target impacts if risks are appropriately assessed. These findings provide a foundation for further research and policy evaluation in support of pollinator conservation under changing environmental and political conditions.

**Supplementary Information:**

The online version contains supplementary material available at 10.1038/s41598-026-53025-1.

## Introduction

Pollinators are globally recognised as essential for both agricultural productivity and the resilience of natural ecosystems^1^. In Europe, where insect pollination supports high-value crops, the annual contribution of pollinators is between €5–15 billion^2^. Beyond crops, pollinators are critical for maintaining diverse plant communities and supporting broader biodiversity and ecosystem services^3–5^.

Worryingly, declines in insect populations and species extinctions have been recorded across most habitats in all regions of the world^6^. Increasing evidence indicates that these declines in the abundance and diversity of various pollinator taxa have occurred at local, regional, and global scales^4^. The current rate of decline for terrestrial insects is estimated to be 9% per decade^7^. Within the European Union, 9.1% of bee species are classified as at risk of extinction^8^.

A range of anthropogenic factors have been identified as threats to pollinators and drivers of declines^1,9^. These include habitat loss and fragmentation, pesticide use, parasites and pathogens, invasive species, and climate change^9–11^. These anthropogenic stressors can work individually but also together in additive and synergistic interactions that intensify the negative impacts on pollinators^11–14^.

Both practice and policy are needed to mitigate these stressors^15^. Policy responses in Europe, and around the globe, have begun to address some of these issues^16^. For example, the EU Pollinators Initiative, launched in 2018, is the European Union’s first coordinated framework to address pollinator decline. It focuses on improving knowledge of pollinator populations and drivers of decline, tackling the causes of decline, and engaging society and stakeholders^17^. These objectives have been reinforced through the Farm to Fork Strategy and the EU Biodiversity Strategy for 2030, which place greater emphasis on habitat restoration, reducing pesticide dependency, and enhanced monitoring^17,18^. Multiple countries within Europe have also developed national pollinator strategies, yet most remain focused on well-established existing pressures rather than novel threats.

To safeguard pollinators in the face of future changes, conservation efforts must anticipate emerging threats and opportunities^10^. Horizon scanning provides a structured approach to identify such nascent challenges and opportunities before they escalate^19^. When used effectively, it can inform timely policy actions that are preventive rather than reactive. Previous horizon scans have successfully identified key emerging issues and opportunities for biodiversity conservation^20^. Here, we convened a diverse group of experts across disciplines and regions to conduct a horizon scan focused on the future of wild pollinators in Europe. We followed a standardised horizon scanning methodology^21^, which was used for the first global horizon scan of pollinators and pollination^10^. We aim to highlight topics that may soon become critical for pollinator conservation, positively or negatively, but have yet to attract widespread attention from researchers and decision-makers.

## Methods

**Fig. 1:**
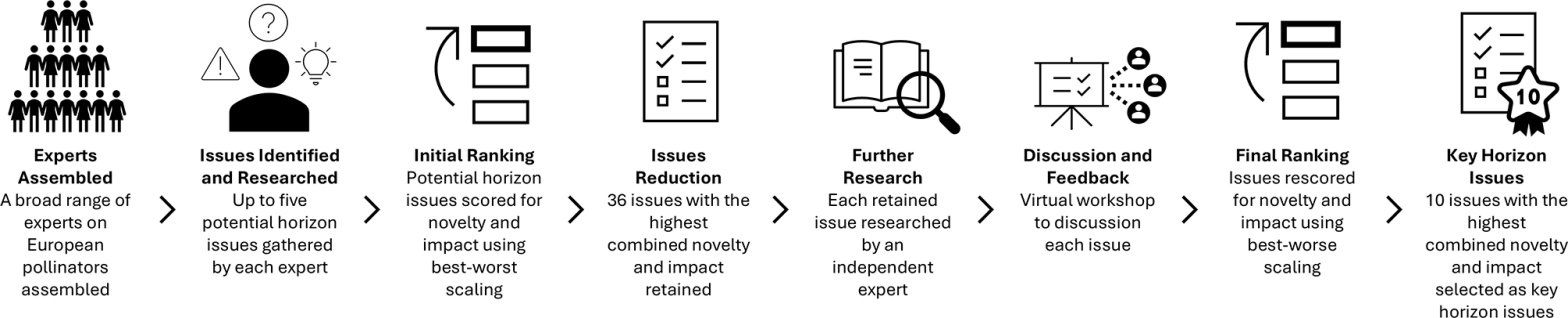
Overview of the horizon scan used to identify 10 emerging horizon issues.

To conduct the horizon scan, we followed an approach based on the Delphi method (described below)^21^ (Fig. 1). Our horizon scanning group was comprised of 18 experts in European pollinators (the authors, and one additional expert who withdrew after the completion of the study from the author list due to a conflict of interest; as this withdrawal occurred at the end of the process, it had no impact on the study itself), balanced across taxonomic expertise, gender, career stage, and geographic knowledge. Experts were drawn from NGOs, policy organisations, industry, research institutes, and universities.

### Selecting issues

Each expert in the horizon scanning group gathered up to five potential horizon issues by (i) brainstorming with or canvasing their colleagues (both in and outside the pollinator community), (ii) exploring social media and online communities, (iii) scanning news and media outlets, blogs, podcasts, and government/NGO reports, and (iv) examining recent scientific literature. Where the Horizon Scan experts had conversations with people in their professional networks, the contacts were aware that this was for a Horizon Scan and gave informed consent to their ideas being used within the Scan. This process followed standard practice for Horizon Scans, which do not fall under institutional ethics committees, as they are neither surveys nor experimental in nature. In total, 41 people (see ‘Acknowledgements’) were consulted as part of this process.

Our search focused explicitly on issues that were poorly known and might have a significant positive or negative impact on wild pollinators in Europe between 2025 and 2035. Issues were submitted using a template that enabled consistency and clarity, for each issue, on why it was novel and why it might have a major impact, as well as the source of the issue (Table S1).

In total, 97 potential issues were submitted. These were condensed to 79 issues (combining issues that had been proposed by multiple experts) (Table S1). This list was scored for novelty and impact using best-worse scaling on the OpinionX platform (opinionx.co). Best-worse scaling is a quantitative methodology that enables prioritisation of issues (reviewed by^22,23^). Issues with lower score values had higher priority. Issues were presented to horizon scanners in groups of six, based on the total number of issues and following the analysis of Hollis (2020)^24^. The overall priority of an issue was determined by its novelty and impact. For scoring, the novelty of an issue was defined as (i) previously unidentified as an opportunity or threat to wild pollinators, or (ii) only just emerged on the scene, or (iii) a new manifestation of a previously identified opportunity or threat. The impact of an issue over the following decade was defined as high if it was judged likely to have a large positive or negative effect on wild pollinator populations across large areas of Europe. Issues that were likely to have a large effect but in a geographically restricted area, or a smaller effect but across a large geographical area, were, by definition, of lower impact. After scoring, the novelty and impact scores (where lower score values indicated higher novelty or impact) were multiplied^25^ to enable overall ranking of issues (Table S1).

### Refining to a shortlist of priorities

After overall ranking, 36 issues with the lowest scores (highest novelty or impact) were retained. Experts were given the opportunity to propose and argue for the retention of any dismissed issues, but no issues were proposed. Each expert was then given two issues for which they acted as lead researcher, and two issues where they acted as secondary researcher. Consequently, each issue had two experts allocated to it, neither of whom had originally proposed it. Experts were asked to conduct an in-depth assessment of the novelty and impact of their allocated issues. A template was provided to enable experts to review (according to the evidence base) and report on the novelty of issues across the following five axes: (i) has this idea been proposed previously in horizon scans or reviews?, (ii) has it emerged recently as an idea?, (iii) has work already been done that assesses the impact of this idea on wild pollinators in Europe?, (iv) is it poorly known?, (v) are its potential impacts unexplored? Similarly, scanners reported on the potential impact of issues across three axes (i) is it likely to have a positive/negative impact over the next 5–10 years on wild pollinators in Europe?, (ii) is it likely to positively/negatively impact a large number of wild pollinator species or populations?, (iii) is it likely to positively/negatively impact wild pollinators across a large proportion of Europe?

All reviews were collated and provided to all experts before an online workshop on 1st April 2025. During the workshop, each issue was presented by its primary researcher, and then discussed by the group. One expert could not attend the workshop, so recordings of the workshop and all materials were supplied to all experts directly after the workshop. Experts were then tasked to independently re-score these 36 issues within a week of the workshop. Scoring was as described above, except that issues were presented in groups of five, due to the reduced number of issues being scored^24^. Again, a multiplicative approach was used to generate a final ranked list of issues (Table S2).

Three issues (“Decline in landscape diversity”, “Direct impacts of climate change and extreme weather events”, “Intensification of land use”) were removed from the final priority list, despite their high overall and impact ranking (Table S2). During the workshop, the scanning group agreed that all of these issues were well-known, and had already been either heavily researched or covered in previous horizon scans or multiple reviews. Therefore, these issues do not fit with the aims of a horizon scan. The ten issues presented in this report had the lowest scores (therefore highest novelty/highest impact) from this modified priority list. Following the previous global horizon scan for pollinators and pollination^10^, and taking advantage of a natural break in scores, we present these issues divided into High Priority (HPI) and Secondary Priority issues (SPI).

## Results

**Fig. 2:**
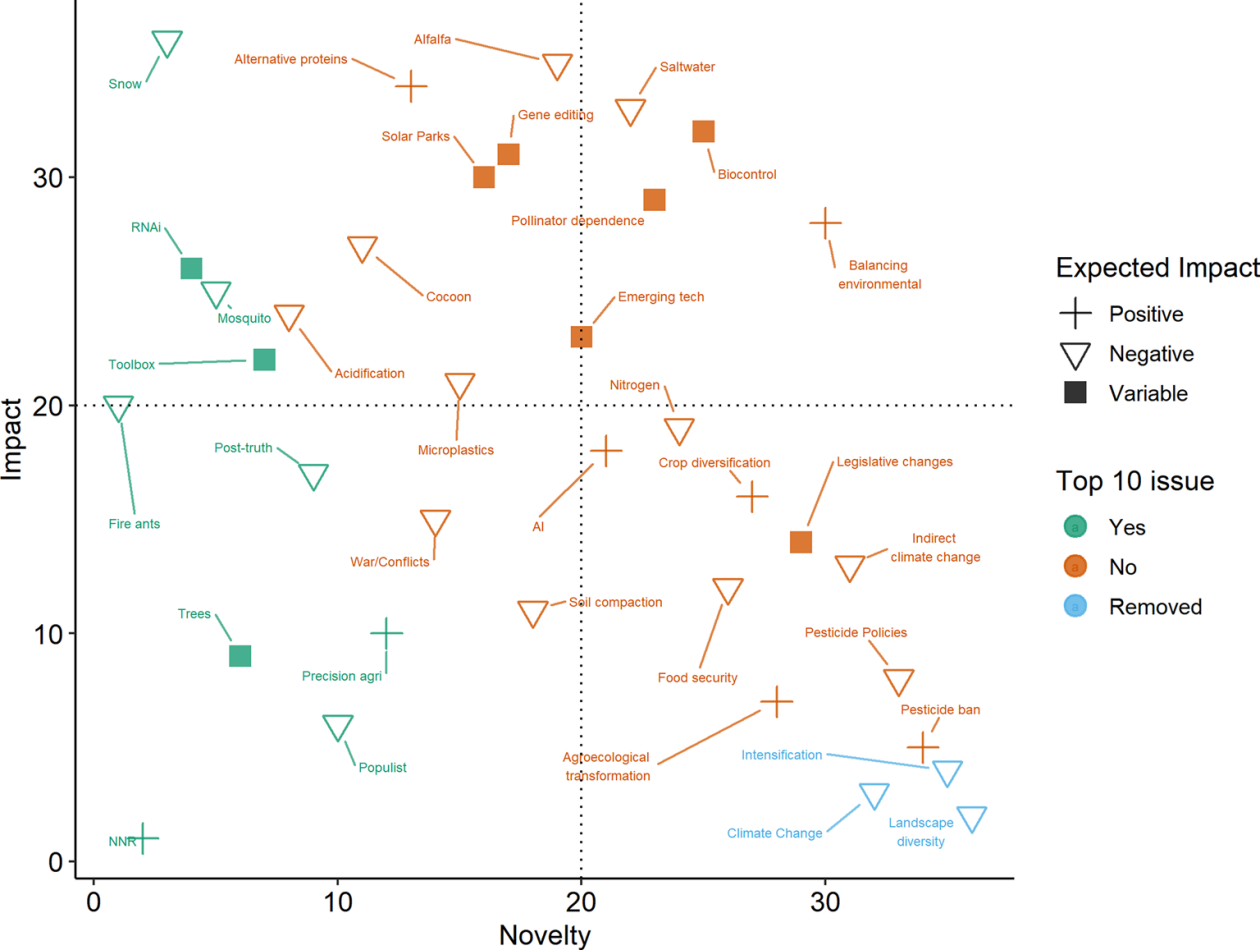
The novelty and impact scores for all issues included in the final round of voting. The majority of the top 10 scoring issues fall within the bottom quartile of the graph. The top 10 horizon issues are shown in green, those that did not make the top ten in orange, and the three issues removed (see Methods) are shown in blue. The point shapes indicate the expected impact direction: positive (+), negative (black filled inverted triangle), or variable (black filled square).

Using a best-worst scaling method, we identified 79 initial potential issues, which were reduced to four high-priority issues and six secondary-priority issues (Fig. 2). These are presented below in descending order of priority.

### HPI 1: Nature Restoration Regulation‚ a once in a generation opportunity for wild pollinators

The European Union’s Nature Restoration Regulation (NRR) entered into force in August 2024^26^. It is the most significant piece of nature legislation in the EU in decades, since the adoption of the Birds Directive (1979) and Habitats Directive (1992). It encompasses a broad range of legally-binding biodiversity commitments to restore ecosystems, habitats, and species across the EU’s land and sea areas. Previous EU initiatives to protect wild pollinators have had significant gaps in management and lacked effective mechanisms to enable protection^27^. However, the NRR is a global first for legislation, with mandatory targets and obligations for pollinator restoration across the EU. Article 10 of the NRR requires Member States to implement measures to reverse the decline of pollinator populations by 2030 and achieve an increasing trend from 2030 until satisfactory levels are achieved^26^. Within the NRR, restoration targets extend beyond semi-natural habitats to agricultural ecosystems and urban areas. Therefore, the positive impacts and opportunities of the NRR may exceed any previous environmental policy, making the NRR a unique and world-leading opportunity for pollinator conservation.

Alongside nature restoration targets, the NRR also requires Member States to collect monitoring data on pollinator abundance and species richness. This would be the first formal supra-national-scale monitoring scheme for pollinators, presenting a unique opportunity for assessing both pollinator trends and the efficacy of conservation interventions^28^.

The NRR represent a unique policy for advancing pollinator conservation. It has the potential to have significant positive impacts on pollinators over the next decade. However, the extent of this impact depends on the implementation of well-considered interventions and the commitment to the non-trivial task of large scale pollinator monitoring.

### HPI 2: New invasive alien species *Solenopsis invicta* (fire ant) reported in Europe

The fire ant, *Solenopsis invicta*, is classified among the most damaging invasive alien species, with significant impacts on ecosystems, agriculture, and human health^29^. They are ranked as the fifth most costly invasive species globally^30^. Fire ants attack, kill, and consume invertebrates, including butterfly larvae, that are unable to defend themselves or escape^31^. In the United States, fire ants are reported to decrease floral visitation and foraging time as a result of aggressive behaviour and the threat of attack^32^. They have been recognised as an invasive species in Europe by both IPBES and the European Food Safety Authority^29,33^, with the first mature population record in Sicily, Italy in 2023^34,35^. Although specific evidence of their impact on European pollinators is currently lacking, generalist predation is recognised by IPBES as a major threat to pollinator populations^1^, and in the US, removal of fire ants increased butterfly abundance^36^. In Europe, under current environmental conditions, fire ants could establish in approximately 50% of urban areas and 7% of Europe^35^. Fire ants may exhibit greater cold tolerance than expected based on their subtropical origin, suggesting that cooler habitats, including higher altitudes and more northern regions, could also be at risk^37^. Modelling studies predict that the potential distribution of fire ants overlaps substantially with areas of high pollinator diversity and endemism across southern Europe^38^. Additionally, climate change and increased global trade may facilitate the spread and population growth of fire ants in Europe^39^. Consequently, fire ants may pose a particularly significant threat to regions of high pollinator diversity. However, it is important to note that the scale of their impact will depend on their rate of spread and the effectiveness of control measures implemented to limit their expansion.

### HPI 3: Tree planting initiatives displacing existing pollinator-rich habitats

Tree planting, driven by multiple needs, including timber, carbon storage, and biodiversity, is now embedded in legislation, policy initiatives, voluntary programmes, and best practice guidelines across Europe^40^. The EU Biodiversity Strategy for 2030 alone commits to planting at least three billion additional trees^41^.

EU-wide guidelines for tree species selection typically focus on maximising tree establishment, resilience, and carbon outcomes. They do not provide clear recommendations for selecting species that support other aspects of biodiversity, including pollinators^40^. For example, tree planting may still displace more valuable habitats for pollinators, such as semi-natural grasslands^42,43^ and shrublands^44^. Tree species selected mainly for fast growth and carbon storage, such as *Eucalyptus* spp., often offer few benefits and may even harm wildlife^45^.

While tree planting for net zero targets was initially proposed as a threat to pollinators, further discussion, as part of the horizon scanning process, also identified a significant opportunity for wild pollinators. Planting a diverse mix of species that support wildlife, such as wild cherry and willow, can enhance floral resources and improve habitat structure^40,45^. Pollinator focused charities (e.g. the Bumblebee Conservation Trust) and national guidelines, such as the Tree Species Guide for UK Agroforestry Systems^45^, include recommendations for supporting biodiversity. However, these are often underutilised, and constrained by limited awareness and management objectives that prioritise carbon storage and timber production over ecological outcomes. As Europe accelerates large-scale tree planting and more organisations offer incentives, training, and advice, planting schemes must be planned with biodiversity outcomes, including wild pollinators.

### HPI 4: Shifts to populist political parties impact biodiversity agenda

Electoral gains by populist parties across Europe have raised concerns about the weakening of environmental and biodiversity protections^46^, as a result of policy rollbacks in several countries. For instance, in Sweden, the governing coalition proposed removing the mandatory integrated pest management (IPM) measures required under the Sustainable Use of Pesticides Regulation (SUR) of the EU (which has now been withdrawn)^47^. Alongside this, they have promoted crop-specific pesticide rules which, when combined, could result in no net reduction in pesticide use^48^. In the Netherlands, populist parties have delayed nitrogen reduction targets, pushing deadlines back from 2030 to 2035 which defies court rulings and undermines established environmental commitments^49^.

This trend also threatens pollinator-friendly measures at the EU level. Wider public protests that reflect concerns within the agricultural sector (e.g., French farmers protesting against the Nature Restoration Regulation) have been seized upon by populist parties to position themselves as defenders of rural interests against environmental regulation^50^. With polling showing rising support for these parties, the risk to EU environmental cohesion is growing^51^. As the political balance shifts, future elections will shape the European Commission’s policy priorities, defining the future of green transition in agriculture. A continued focus on increasing food production, securing raw materials and energy, and expanding industrial and agricultural development could weaken environmental protection through increasing habitat destruction in unprotected areas and contradict the current provisions of the sustainable used of pesticides directive (SUD)^52^. The adverse consequences for pollinators across Europe could be both direct and widespread.

### SPI 1: RNAi-based pesticides: a magic bullet?

RNA interference (RNAi)-based pesticides are a novel pest management strategy^53^. Their sequence-guided mode of action allows highly specific targeting of pest species while leaving non-target species, such as pollinators, unaffected^54–57^. In 2023, the first commercial RNAi product was released on the market in the USA^58^. Although the timeline for release in Europe is unknown, at least one firm has publicly stated that it is already conducting open field trials on potato crops in European countries^59^. If RNAi-based pesticides appear on the market and are effective, they could be applied across large areas throughout Europe, providing a significant opportunity for reducing pesticide use in European agriculture, with subsequent benefits to wild pollinators across Europe.

Initial laboratory studies of RNAi pesticides show high sequence specificity^60^ and a promising lack of effects on survival and adult emergence in honey bees^54^. However, several studies, including on honey bees, have reported unintended adverse effects of dietary double stranded RNA (dsRNA) in species that are closely and distantly related to the target pest species^61–64^. It has also been suggested that non-specific dsRNAs could affect non-target organisms via processes such as immune stimulation or via the saturation of the RNAi machinery^65^, although evidence for this is lacking. The dsRNA within RNAi pesticides degrades rapidly over time (e.g. in 14–30 h in sandy soil^66^, reducing the scope for negative impacts on non-target organisms. However, as a result, RNAi pesticides will likely be formulated with stabilisers designed to reduce degradation, some of which themselves pose an already identified threat to pollinators^67^. Consequently, the risk assessment and management of RNAi-based pesticides will determine whether they pose a threat to wild pollinators or provide an opportunity for their conservation.

### SPI 2: Artificial snow and plant-pollinator interactions

Temperatures are rising worldwide, and artificial snow is increasingly used to maintain snow cover^68^. Within ski resorts, data for 2018 shows that 25–87% of total snow area has some artificial snow cover^69^. Artificial snow is predominantly used in Alpine regions, however, it is also applied in other mountainous areas in Europe as well as for cross-country skiing. Though good data are lacking, multiple sources suggest that the use of artificial snow will increase and could impact over 2000 ski resorts in Europe^70^.

Artificial snow possesses different physical and chemical properties compared to natural snow, leading to denser and deeper snow cover as well as delayed snow melt^71^. Its use can change vegetation composition, favouring nutrient- and moisture-demanding species (e.g.,^72^), and impact ground-dwelling invertebrates (e.g.,^73^). To date, no studies have directly examined the impacts of artificial snow on pollinators. Research, albeit not on artificial snow, has shown that delayed snow melt does alter plant-pollinator interactions^74^. In contrast, snow cover and melt timing are unlikely to affect hibernation success in bumblebees^75^. Nevertheless, given its effects on plant communities, indirect impacts on pollinator populations and plant-pollinator interactions are likely. Artificial snow also contains surfactants and bacterial compounds^76^ with potential additional impacts on pollinator health, however, this remains unexplored. Nevertheless, the spatial extent of artificial snow’s threat is limited in comparison to other issues in this Horizon Scan.

### SPI 3: Precision applications: innovative crop protection and fertilising equipment enabling significant reduction of pesticide use and related exposure of wild pollinators, for both conventional and biopesticides

Pesticide exposure is a well-known driver of pollinator declines in Europe^1,11^. Reductions in pesticide use have featured in European legislation such as the European Commission’s Green Deal, which aimed to reduce pesticide use by 50% in European agriculture by 2030^77^. One method to reduce pesticide use, without biochemical innovations, is to develop more precise and effective application methods^78,79^. Precision application has been described as “right practice at the right location and time, and at the right intensity”^80^. This application method would, in theory, reduce the amount and number of pesticide sprays without compromising pest management. Pesticide reduction achieved through directed sprays on weeds, compared to broadcast spraying, is significant and can reach 90% with the use of ultra precision sprayers^81^. This could significantly reduce pesticide exposure for pollinators. However, the capacity to target within-field low-fertility patches with high-precision fertiliser application devices could also result in more homogenous crops that would offer less resources to pollinators than they currently do. The consequences of precision agriculture for pollinators have not been quantified and require research. Although precision agriculture is not a new concept, its adoption and investigation remain limited by the cost of the most recent equipment and the lack of recognition in the regulatory process^82–85^. One potential barrier to its uptake is the challenge of monitoring crops to determine the precise timing and location for crop protection measures, a task that can be labour-intensive and requires good access to data networks^82^. However, advances in remote sensing and artificial intelligence are poised to improve the feasibility of precision applications^86–88^. As these technologies develop, the potential of precision agriculture to reduce pesticide exposure for pollinators may soon be realised.

### SPI 4: Fumigation/spraying against tiger mosquito (*Aedes albopictus*) affects wild pollinators and their habitats

With rising temperatures in Europe, the continent is becoming increasingly suitable for invasive species such as the Asian tiger mosquito, *Aedes albopictus*, which is already present in 16 countries^89^. Under projected climate change, more areas could develop favourable conditions for this species^90^. Therefore, the distributional range of tiger mosquitoes in Europe is likely to increase^90^.

While tiger mosquitoes do not pose a direct threat to wild pollinators, they may present an indirect threat^91,92^. Due to their impact on human health as disease vectors, control strategies have been and are being implemented to control their populations^93^. These strategies include the use of broad-spectrum pesticides^94^. The European Centre for Disease Prevention and Control suggests that aerial sprays of insecticide mixtures can be undertaken as exceptional measures (e.g., in France in 2005-2006^95^). However, the use of aerial sprays or fumigation with broad-spectrum pesticides will also affect non-target organisms, including pollinators, through aerial exposure or uptake from soil or flowers^96,97^. Despite these pesticides being regulated, assessments have not been conducted for the application of aerial sprays and mixtures of insecticides as used in mosquito control. Consequently, mosquito management could lead to widespread exposure to multiple insecticides for wild pollinators. The spread of tiger mosquitoes within Europe may also indirectly affect the presence of stagnant water, such as ponds and bird baths, which can be eliminated due to concerns surrounding mosquitoes. This could impact populations of hoverflies that require stagnant water for development^98^. The risk posed by tiger mosquitoes may be particularly concentrated in urban areas, which can be important for pollinator biodiversity^99,100^.

### SPI 5: Post-truth era challenges to pollinator conservation

The concept of misinformation and declining trust in science affecting conservation efforts has recently gained attention^101^. Coates & Sandroni (2023), for example, highlighted the rise of post-truth politics as a significant challenge to environmental conservation, noting that misinformation can undermine policy implementation^101^. Socio-psychological factors, beyond mere knowledge, significantly influence individuals’ willingness to engage in pollinator conservation actions^102,103^. In a socio-cultural landscape dominated by oversimplified narratives, nuanced messages about insect population trajectories and management strategies struggle to gain traction. For example, emerging positive trends in wild pollinator populations may be twisted into claims that ‘the problem is solved’.

Post-truth challenges to pollinator conservation operate both top-down (around policy) and bottom-up (members of the public and social media). Although science communication has become a cornerstone of conservation strategy over the past decades^104^, mitigating the spread of post-truth misinformation may exceed the capacity of researchers without changes in science education and practice^105^, with subsequent negative impacts on wild pollinators.

### SPI 6: Development of adapted risk assessment toolbox for new crop protection mode of actions (e.g., biopesticides)

Current use of plant protection products in Europe, and globally, is governed by risk assessment systems designed around chemical active ingredients^106^. However, as the agricultural sector in Europe shifts towards more sustainable practices, crop protection products now include a range of biopesticides with new modes of action, from semiochemicals to natural substances, microorganisms, peptides, RNAis (see SPI 1 above), or antibodies^107^. The introduction of biopesticides has highlighted the limitations of existing risk assessment tools, prompting discussions on developing new methodologies^106^. There is consensus in the scientific community that the testing approaches developed for chemical substances may not be appropriate or effective for the new biopesticides^106,108–110^. New crop protection methods often have novel or complex modes of action, and we lack the testing protocols needed to understand the full range of risks^109^. Further, there is greater uncertainty surrounding the outcomes of novel mechanisms in the range of conditions where these pesticides would be used.

Appropriate assessment and subsequent rollout of new crop protection mechanisms could present a significant opportunity to relieve the pressure placed on pollinators by current pesticide exposure. Any benefit for pollinators will be dependent on the development of appropriate risk assessment tools for new crop protection mechanisms.

## Discussion

**Fig. 3:**
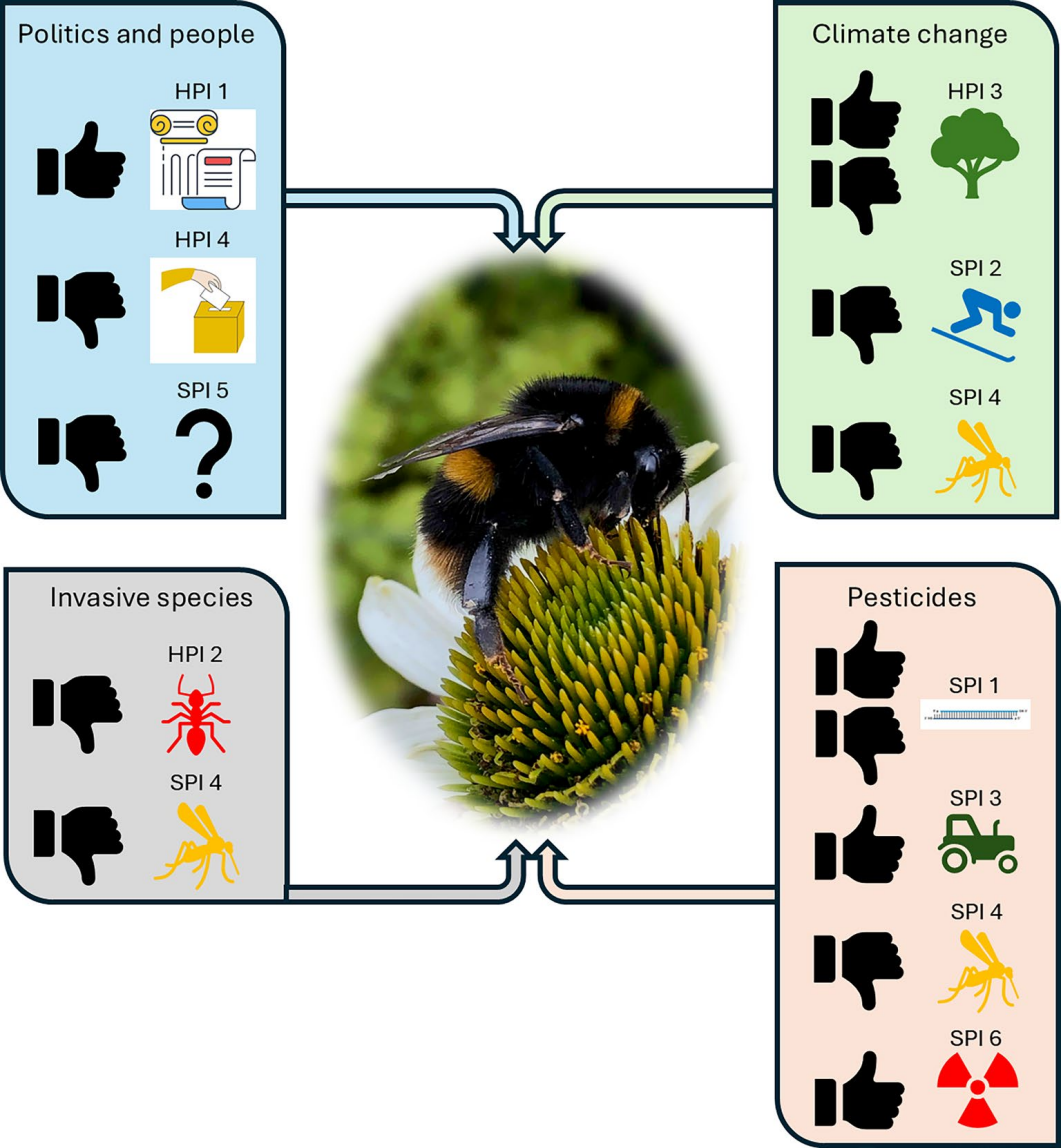
A schematic grouping the top ten identified Horizon Issues by theme (politics and people, climate change, invasive species, and pesticides), indicating potential positive (thumbs up), negative impacts (thumbs down) or potentially variable/multidirectional impacts (both thumbs up and down). Individual Horizon Issues are labelled with their representing issue rank code and accompanied by pictograms illustrating their focus.

In a dynamic world, new opportunities and threats for wild pollinators are constantly emerging. Here we have identified a suite of Horizon Issues that may have both positive and negative impacts on wild pollinators in Europe over the next decade (Fig. 3). These add to already well-established pressures from landscape simplification and habitat loss, pests and pathogens, climate change and intensive land management^1^. Importantly, and for the first time, actual legislation was identified as the most important opportunity for pollinators (HPI 1). While the value of legislation for nature conservation more broadly is well-known, the Nature Restoration Regulation of the EU is a global first, targeting wild pollinator biodiversity at a continental scale. As such, it may act as a model for action in other regions by illustrating how specific pollinator restoration and monitoring targets within a large scale biodiversity policy can accelerate the implementation of wild pollinator conservation measures. This impact is absolutely reliant on the implementation of this legislation. Consequently, it is concerning that our Horizon Scan identified two other issues – populist politics (HPI 4) and post-truth discourse (SPI 5) – that could actively mitigate the effective rollout of NRR actions, generally and specifically concerning pollinators. It is incumbent upon conservation actors – be they scientists, sociologists, psychologist, NGOs, industry, or politicians – to develop strategies and mechanisms that effectively combat the threats of populism and post-truth discourse to wild pollinator conservation.

A suite of our identified Horizon Issues encompass novel invasive species (HPI 2), novel aspects of pesticides (SPI 1, 3, 6), or their interactions (SPI 4). Invasive species are one of the greatest threats to biodiversity^1,111^, but their identification as threats to wild pollinators has previously been largely limited to emerging parasites and pathogens^10^. The impacts of many non-parasitic invasive species on pollinators are indirect, context-dependent, and mediated through complex ecological interactions, which complicates their detection and quantification^112^. Similarly, pesticides have previously been identified as horizon issues with negative impacts on pollinators^10^, and so it is exciting that the three pesticide-related Horizon Issues identified here can actually provide opportunities for enhancing wild pollinator health.

While future Horizon Scans will inevitably identify new and emerging opportunities and threats for wild pollinators, we believe that the Horizon Issues identified here provide significant opportunities for action and research that will enhance wild pollinators in Europe, and beyond, in years to come.

## Supplementary Information

Below is the link to the electronic supplementary material.

Supplementary Material 1

## Data Availability

All data used in this study are provided in the supplementary materials.
